# Present and future of the diagnostic work-up of multiple sclerosis: the imaging perspective

**DOI:** 10.1007/s00415-022-11488-y

**Published:** 2022-11-24

**Authors:** Massimo Filippi, Paolo Preziosa, Douglas L. Arnold, Frederik Barkhof, Daniel M. Harrison, Pietro Maggi, Caterina Mainero, Xavier Montalban, Elia Sechi, Brian G. Weinshenker, Maria A. Rocca

**Affiliations:** 1 Neuroimaging Research Unit, Division of Neuroscience, IRCCS San Raffaele Scientific Institute, Via Olgettina, 60, 20132 Milan, Italy; 2 Neurology Unit, IRCCS San Raffaele Scientific Institute, Milan, Italy; 3 Neurorehabilitation Unit, IRCCS San Raffaele Scientific Institute, Milan, Italy; 4 Neurophysiology Service, IRCCS San Raffaele Scientific Institute, Milan, Italy; 5 Vita-Salute San Raffaele University, Milan, Italy; 6 NeuroRx Research, Montreal, QC Canada; 7 McGill University, Montreal, QC Canada; 8 Department of Radiology and Nuclear Medicine, Amsterdam UMC, Vrije Universiteit, Amsterdam, Netherlands; 9 Queen Square Institute of Neurology and Centre for Medical Image Computing, University College London, London, UK; 10 Department of Neurology, University of Maryland School of Medicine, Baltimore, MD USA; 11 Department of Neurology, Baltimore VA Medical Center, Baltimore, MD USA; 12 Department of Neurology, Cliniques Universitaires Saint Luc, Université Catholique de Louvain, Brussels, Belgium; 13 Department of Neurology, Centre Hospitalier Universitaire Vaudois (CHUV), Lausanne, Switzerland; 14 Department of Radiology, Athinoula A. Martinos Center for Biomedical Imaging, Massachusetts General Hospital, Boston, MA USA; 15 Harvard Medical School, Boston, MA USA; 16 Department of Neurology, Cemcat, Hospital Vall d’Hebron, Autonomous University of Barcelona, Barcelona, Spain; 17 Department of Medical, Surgical and Experimental Sciences, University of Sassari, Sassari, Italy; 18 Department of Neurology, University of Virginia, Charlottesville, VA USA

**Keywords:** Multiple sclerosis, Magnetic resonance imaging, Diagnosis

## Abstract

In recent years, the use of magnetic resonance imaging (MRI) for the diagnostic work-up of multiple sclerosis (MS) has evolved considerably. The 2017 McDonald criteria show high sensitivity and accuracy in predicting a second clinical attack in patients with a typical clinically isolated syndrome and allow an earlier diagnosis of MS. They have been validated, are evidence-based, simplify the clinical use of MRI criteria and improve MS patients’ management. However, to limit the risk of misdiagnosis, they should be applied by expert clinicians only after the careful exclusion of alternative diagnoses. Recently, new MRI markers have been proposed to improve diagnostic specificity for MS and reduce the risk of misdiagnosis. The central vein sign and chronic active lesions (i.e., paramagnetic rim lesions) may increase the specificity of MS diagnostic criteria, but further effort is necessary to validate and standardize their assessment before implementing them in the clinical setting. The feasibility of subpial demyelination assessment and the clinical relevance of leptomeningeal enhancement evaluation in the diagnostic work-up of MS appear more limited. Artificial intelligence tools may capture MRI attributes that are beyond the human perception, and, in the future, artificial intelligence may complement human assessment to further ameliorate the diagnostic work-up and patients’ classification. However, guidelines that ensure reliability, interpretability, and validity of findings obtained from artificial intelligence approaches are still needed to implement them in the clinical scenario. This review provides a summary of the most recent updates regarding the application of MRI for the diagnosis of MS.

## Introduction

A diagnosis of multiple sclerosis (MS) requires a symptomatic demyelinating syndrome with objective neurologic findings, the demonstration of a pathological process disseminated in space (DIS) and time (DIT) and the exclusion of alternative conditions [1].

Recently, the enhanced characterization of clinical and radiologic features associated with different inflammatory demyelinating disorders of the CNS [2, 3] and improvements in neuroimaging and laboratory technologies have contributed to the refinement of the diagnostic work-up of patients with a suspicion of MS [4]. Subsequent iterations of the McDonald criteria have defined evidence-based imaging features typical of MS, facilitating earlier fulfillment of the diagnostic criteria for MS [1].

Since a specific MS biomarker is not available, simplifications and easier fulfilment of the diagnostic criteria (e.g., requiring fewer MRI lesions and substituting detection of intrathecal immunoglobulin G [IgG] synthesis for DIT) may increase the risk of MS misdiagnosis [5, 6]. These considerations have prompted extensive research in the field of neuroimaging to identify novel MRI features more specific to MS. Moreover, the use of artificial intelligence (AI) has been recently suggested as a new promising tool for MS clinical practice [7].

An international meeting was held on the 3rd of November 2021, which involved neurologists and (neuro)radiologists with expertise in MS and its mimics (see Acknowledgments for details) to summarize the most recent applications of MRI in the MS diagnostic work-up but also possible future innovations. The key aspects discussed in the meeting included the current evidence regarding the clinical application of the 2017 McDonald criteria, promising novel markers to improve accuracy of diagnosis, and the potential contribution of AI for MS diagnostic work-up.

Experts provided a summary related to each topic (see Table 1 for search strategy and selection criteria). A group consensus was reached during the meeting and summarized in a first draft, which was circulated among the speakers and additional experts in the field for critical discussion and revision.

**Table 1:** Search strategy and selection criteria

Sources	Pubmed (https://www.ncbi.nlm.nih.gov/pubmed)
Period of time covered	From January 1979 until October 2022
Search terms	“Artificial intelligence”, “Chronic active lesions”, “Cortical lesions”, Deep learning”, “Diagnostic Criteria”, “Differential Diagnosis”, “Inflammation”, “Iron Rim Lesions”, “Leptomeningeal Inflammation”, “Lesion/s”, “MRI”, “Machine Learning”, “McDonald criteria”, “MOGAD”, “Multiple Sclerosis”, “NMOSD”, “Paramagnetic Rim Lesions”, “Primary Progressive”, “Spinal Cord”, “Subpial Demyelination”, “White Matter”
Selection criteria and review preparation	1. Only papers published in English 2. The final reference list was generated with the consensus of all co-authors of this review on the basis of originality and relevance to the broad scope of this review, with a focus on the most recent articles published in the last three years 3. Experts provided a summary during the meeting of the main findings related to specific topics of the review. For each topic, a group consensus was reached and summarized in a first draft, which was circulated among the co-authors for further critical discussion and revision. The review represents the final conclusions reached by co-authors

This review summarizes the current state-of-the-art and possible future applications of MRI technologies for the diagnostic work-up of MS.

Increasing attention is also given to the diagnosis and prognostication of subjects with brain MRI abnormalities suggestive of MS, but lacking historical accounts of prior demyelinating events (i.e., “radiologically isolated syndrome” [RIS] or “prodromic phase of MS”). This is out-of-scope of the present review and it is described elsewhere [8–10].

## The 2017 McDonald criteria: from statements to clinical use

The diagnosis of MS is primarily based on clinical criteria. Since 2001, MRI has been included in MS diagnostic work-up to support, supplement, or even replace some clinical criteria in excluding differential diagnosis and demonstrating DIS and DIT [1]. In patients with a typical clinically isolated syndrome (CIS) suggestive of MS, from their introduction, subsequent iterations of the McDonald criteria have simplified MS diagnosis, improving sensitivity and preserving accuracy. The last revision of the McDonald criteria (i.e., the 2017 McDonald criteria) [1] included the removal of any distinction between symptomatic and asymptomatic lesions, and the combination of cortical lesions and juxtacortical lesions to expand the concept of juxtacortical involvement. Furthermore, in patients with a typical CIS suggestive of MS, the presence of CSF-specific oligoclonal bands (OCBs) supplants demonstration of DIT (Table 2).

**Table 2:** The 2017 McDonald criteria for diagnosis of multiple sclerosis

Clinical presentation	Clinical presentation	Additional data needed for MS diagnosis
Relapse-onset (CIS)	≥ 2 clinical relapses and objective clinical evidence of ≥ 2 lesions; OR ≥ 2 clinical relapses and objective clinical evidence of 1 lesion and clear-cut historical evidence of a prior relapse involving a lesion in a distinct anatomic location	None
	≥ 2 clinical relapses and objective clinical evidence of 1 lesion	DIS, demonstrated by: A second clinical relapse implicating a different CNS site OR demonstration of DIS by MRI (≥ 1 lesion in ≥ 2 of the following regions: periventricular, cortical/juxtacortical, posterior fossa, spinal cord)
	1 clinical relapse and objective clinical evidence of 2 or more lesions	DIT, demonstrated by: A second clinical relapse OR demonstration of DIT by MRI Simultaneous presence of Gd-enhancing and non-enhancing lesions at any time (including symptomatic lesions) or a new T2 and/or Gd-enhancing lesion on FU MRI irrespective of timing of baseline scan OR demonstration of CSF-specific OCBs*
	1 clinical relapse and objective clinical evidence of 1 lesion	DIS and DIT, demonstrated by: For DIS: A second clinical relapse implicating a different CNS site OR demonstration of DIS by MRI (≥ 1 lesion in ≥ 2 of the following regions: periventricular, cortical/juxtacortical, posterior fossa, spinal cord) For DIT: A second clinical relapse OR demonstration of DIT by MRI Simultaneous presence of Gd-enhancing and non-enhancing lesions at any time (including symptomatic lesions) or a new T2 and/or Gd-enhancing lesion on FU MRI irrespective of timing of baseline scan OR demonstration of CSF-specific OCBs*
Progressive-onset (PPMS)	1 year of disability progression (retrospectively or prospectively determined) independent of clinical relapse	≥ 2 out of 3 of the following criteria: • ≥ 1 T2-hyperintense lesions in ≥ 1 areas in the brain characteristic of MS (periventricular, cortical/juxtacortical or infratentorial) • ≥ 2 T2-hyperintense lesions in the spinal cord, with no distinction between symptomatic or asymptomatic lesions • Presence of CSF-specific OCBs

*CIS* clinically isolated syndrome, *CNS* central nervous system, *DIS* dissemination in time, *DIT* dissemination in time, *Gd* gadolinium, *MRI* magnetic resonance imaging, *MS* multiple sclerosis, *OCB* oligoclonal band, *PPMS* primary progressive multiple sclerosis

*In patients with a typical CIS suggestive of MS fulfilling DIS criteria and with no better explanation for the clinical presentation, the demonstration of CSF-specific OCBs substitutes for the requirement of fulfilling DIT, thus allowing a diagnosis of MS, even if the clinical and MRI findings do not meet the criteria for DIT

Several validation studies in different countries [11–16] showed that the 2017 McDonald criteria have higher sensitivity, lower specificity and similar accuracy compared with the 2010 criteria in predicting the second clinical attack not only in adults, but also in pediatric patients.

In a recent large multicenter study with 785 CIS patients suggestive of MS from 9 European centers, the 2017 vs 2010 McDonald criteria had higher sensitivity (0.83 vs 0.66), lower specificity (0.39 vs 0.60), but similar area under the curve (AUC) values (0.61 vs 0.63) [11].

The inclusion of lesions in the symptomatic region in patients with CIS with a brainstem or spinal cord onset is likely to increase the sensitivity and to decrease specificity, without affecting the accuracy of diagnostic criteria [17]. Although cortical lesion assessment in the diagnostic algorithm of CIS patients has been found to increase specificity [18, 19], the combination of cortical and juxtacortical lesions to define juxtacortical involvement does not substantially influence the performance of diagnostic criteria [17].

In addition to MRI modifications, CSF-derived data also influence the performance of diagnostic criteria since the evaluation of the 2017 McDonald criteria without CSF-specific OCB assessment decreased sensitivity (0.74), increased specificity (0.54), and preserved AUC values (0.64) [11].

Moreover, the 2017 McDonald criteria substantially shorten the time to MS diagnosis, with more CIS patients fulfilling a diagnosis of MS already at the time of the first clinical manifestation and with a single MRI scan [11–13]. The 2017 McDonald criteria shortened the median time to MS diagnosis by 4.6 years compared with the clinical criterion alone and by 10 months compared with the 2010 McDonald criteria (median time to MS diagnosis: 2017 McDonald criteria = 3.2 months) [11]. This earlier diagnosis is possible not only thanks to modifications of MRI criteria, but also to the relevant contribution of CSF-specific OCB evaluation (median time to MS diagnosis: 2017 McDonald criteria without OCBs = 11.4 months) [11].

An earlier MS diagnosis may facilitate earlier treatment. In a study of 1174 patients with CIS suggestive of MS [20], the median times from CIS to MS diagnosis and from CIS to treatment initiation were reduced by 77% and 82%, from the Poser [21] to the 2017 McDonald criteria [1] periods. A significantly lower risk of reaching an Expanded Disability Status Scale (EDSS) score ≥ 3.0 was also found for patients diagnosed with the most recent diagnostic criteria [20].

Up to 15% of MS patients experience a gradual clinical progression from disease onset (i.e., primary progressive [PP] MS) [1]. According to the 2017 McDonald criteria, PPMS can be diagnosed in patients with ≥ 1 year of disability progression independent of clinical relapses who also fulfill at least two of the following three criteria: (1) ≥ 1 lesion(s) in 1 or more topography including periventricular, cortical/juxtacortical, or infratentorial brain regions; (2) ≥ 2 lesions in the spinal cord; and (3) CSF-specific OCBs (Table 2). A recent study with 117 PPMS patients showed that sensitivity (89 vs 85%), specificity (100 vs 100%) and accuracy (91 vs 87%) of the 2017 and 2010 McDonald criteria for progressive- and relapse-onset MS were similar, suggesting to apply a single set of MRI criteria for both relapse-onset and progressive-onset patients [22].

Simplification and liberalization of MS diagnostic criteria raised concerns for an increased risk of misdiagnosis and inappropriate use of treatments [5, 6]. MS misdiagnoses mostly occur due to inappropriate application of the diagnostic criteria in patients with other inflammatory CNS disorders that can meet the DIS and DIT requirements, or erroneous interpretation of MRI abnormalities [5, 6]. To minimize misdiagnosis a careful exclusion of alternative diagnoses is necessary before applying the 2017 McDonald criteria since they should be applied primarily in patients with a typical CIS [1].

To this aim, standardized MRI protocols [23], careful determination of which imaging patterns constitute ‘typical’ or ‘atypical’ MS features and guidelines for a proper interpretation of imaging findings [4] are crucial.

In particular, small-vessel disease caused by chronic hypoxia represent the most common differential diagnosis for white matter (WM) lesions on brain MRI. This may occur as an age-related phenomenon and is found more commonly in smokers, patients with hypertension, diabetes and migraine and in various other vascular disorders [24]. Although often recognizable as small, rounded lesions in the deep WM, when becoming more numerous, they may also be periventricular. For this reason, the magnetic resonance imaging in MS (MAGNIMS) group recommended requiring at least 3 periventricular lesions, especially in elderly patients or those with cardiovascular risk factors [1]. This criterion has been supported also by a recent multicenter study showing that three periventricular lesions improved the specificity and accuracy of the 2017 McDonald DIS criteria, especially in patients with CIS aged ≥ 45 years [11].

Recent studies explored whether the inclusion of optic nerve involvement as a fifth region for DIS fulfilment in CIS patients who had visual-evoked potentials or optic nerve MRI evaluations influenced the performance of the diagnostic criteria [17, 25, 26]. Such an addition slightly improved the diagnostic performance by increasing sensitivity without substantially losing specificity, both in CIS patients with and without optic neuritis as the first manifestation of the disease [17, 25, 26].

Moreover, more distinctive MRI features of MS need to be identified and validated. Among these, the central vein sign (CVS), leptomeningeal enhancement, subpial demyelination, and chronic active lesions have been focus of several recent lines of research and discussion.

## The central vein sign

Several recent studies have shown that the presence of a vein at the center of WM lesions, the so-called ‘CVS’ [27], can improve the differentiation between MS and its mimics [28]. The perivenular nature of MS lesions on ex-vivo histopathology is well known since many years. Today, this MS specific histopathological feature can be detected in-vivo using susceptibility-weighted MRI [27, 28]. The percentage of WM lesions featuring a central vein on susceptibility-weighted MRI is substantially higher in MS compared to other MS mimics [28–30] such as migraine, small vessel disease, inflammatory CNS vasculopathies, Susac syndrome, neuromyelitis optica spectrum disorders (NMOSD), and myelin oligodendrocyte glycoprotein antibody disease (MOGAD).

In MS, most newly developing T2-hyperintense WM lesions or gadolinium (Gd)-enhancing lesions show the CVS. In a 2.8 years longitudinal study, 62/153 (40.5%) MS patients developed new WM lesions and 50 of them (80.6%) showed new lesions with the CVS [31]. Moreover, 159/233 (68.2%) new T2-hyperintense lesions and 92/114 Gd-enhancing lesions (80.7%) had the CVS, especially in MS patients with a younger age and a higher percentage of CVS-positive lesions at baseline [31].

**Fig. 1:**
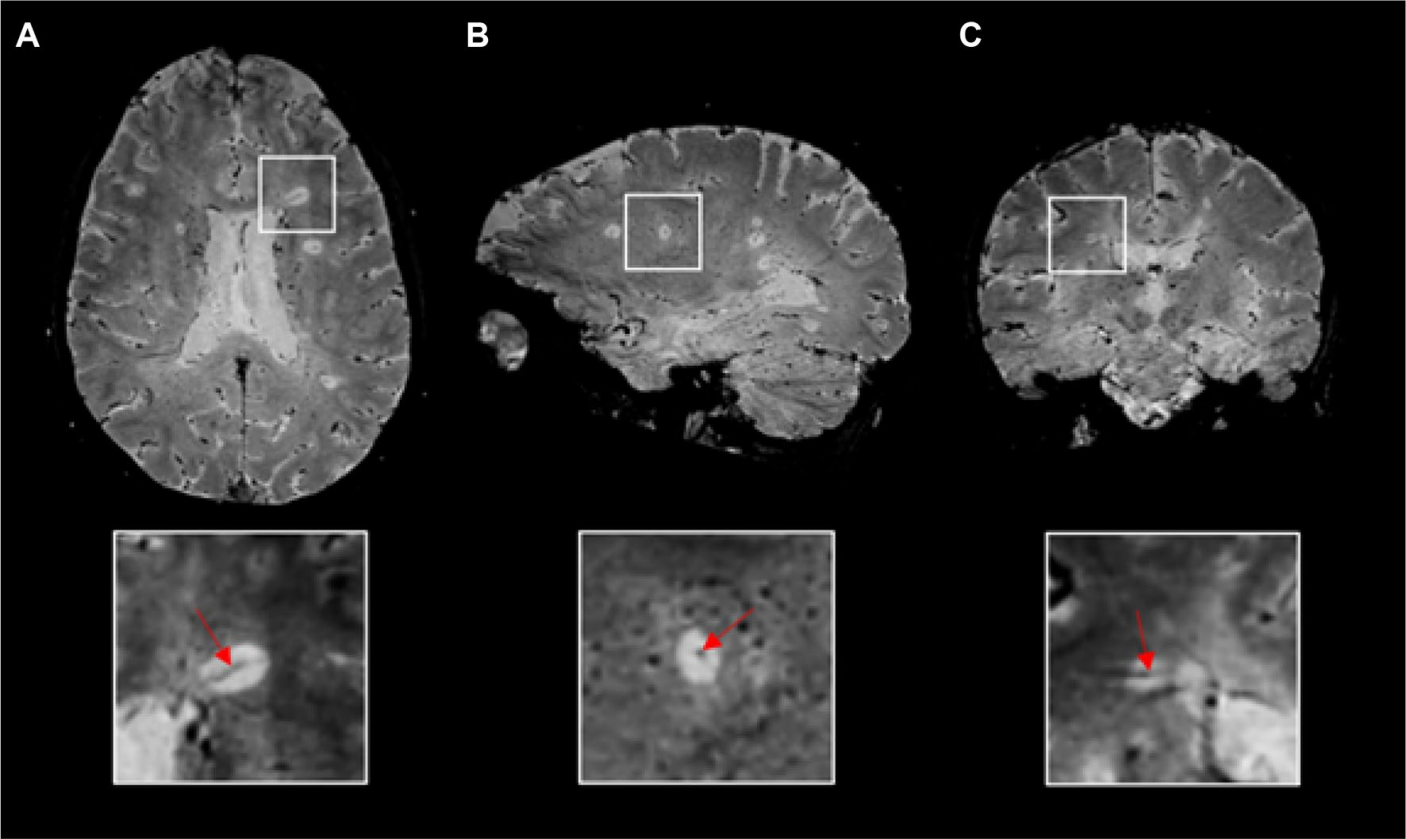
The central vein sign. Representative 3D-EPI T2*-magnitude images in **A** axial, **B** sagittal, and **C** coronal planes acquired at 3T during the injection of gadolinium-based intravenous contrast agent in a 24-years-old relapsing–remitting multiple sclerosis patient. A conspicuous central vein sign is present in the majority of white matter lesions. In the magnified views, a central vein running through the lesion (red arrows) is visible as a hypointense line (axial and coronal planes) or a hypointense dot (sagittal plane). Abbreviations: *3D-EPI* three-dimensional echo planar imaging

Existing evidence from the literature (mainly based on cross-sectional studies) has shown that a 40% CVS positive lesion cut-off can accurately discriminate between MS and other WM diseases [28]. However, the number of CVS-positive lesions detected on a patient’s scan significantly depends on the type of susceptibility sequence used, the MRI field strength and the injection of intravenous Gd (which itself is also paramagnetic, increasing the visibility of veins) [28]. For all these reasons, the percentage of MS CVS lesions and the optimal diagnostic cut-off threshold is higher when using optimized susceptibility-weighted MRI sequences at high and ultra-high field strength (3T and 7T) after Gd injection [28]. Among the different susceptibility-weighted MRI sequences tested so far, the T2*-weighted three-dimensional echo-planar imaging (3D-EPI) has shown the highest capability to detect central veins in MS (Fig. 1) [28, 29]. This is probably due to the high isotropic spatial resolution of the 3D-EPI sequence allowing to detect small intralesional veins in an acceptable scan time. Key challenges preventing the introduction of CVS assessment in clinical practice include: (1) the lack of a standardized imaging protocol (mainly because of the limited availability of optimized MRI sequences from scanner manufacturers), (2) the need of validated CVS-based diagnostic criteria and (3) the need of automated methods to detect the CVS. To overcome these limitations, different CVS-based diagnostic algorithms, including simplified lesion-based diagnostic algorithms (not requiring the analysis of every single lesion) [32], are being tested on a large prospective multicenter setting [33].

Concomitantly, existing statistical and deep-learning based methods for automated CVS detection [34, 35] are being tested on a large scale [33] and efforts to improve their performance are ongoing. Finally, although CVS could potentially be assessed in all scanner manufactures, the different scanner vendors should make optimized and standardized MRI sequences (like the 3D-EPI) available in all MS specialized centers. Given its promising diagnostic performance, the CVS is likely to be incorporated in the MS diagnostic criteria in the near future.

## Chronic active lesions

Pathological studies have revealed that up to 57% of all chronic WM lesions show a peripheral ‘rim’ of iron-laden activated microglia/macrophages associated with ongoing demyelination and axonal loss, around an inactive core without blood–brain barrier damage [36].

**Fig. 2:**
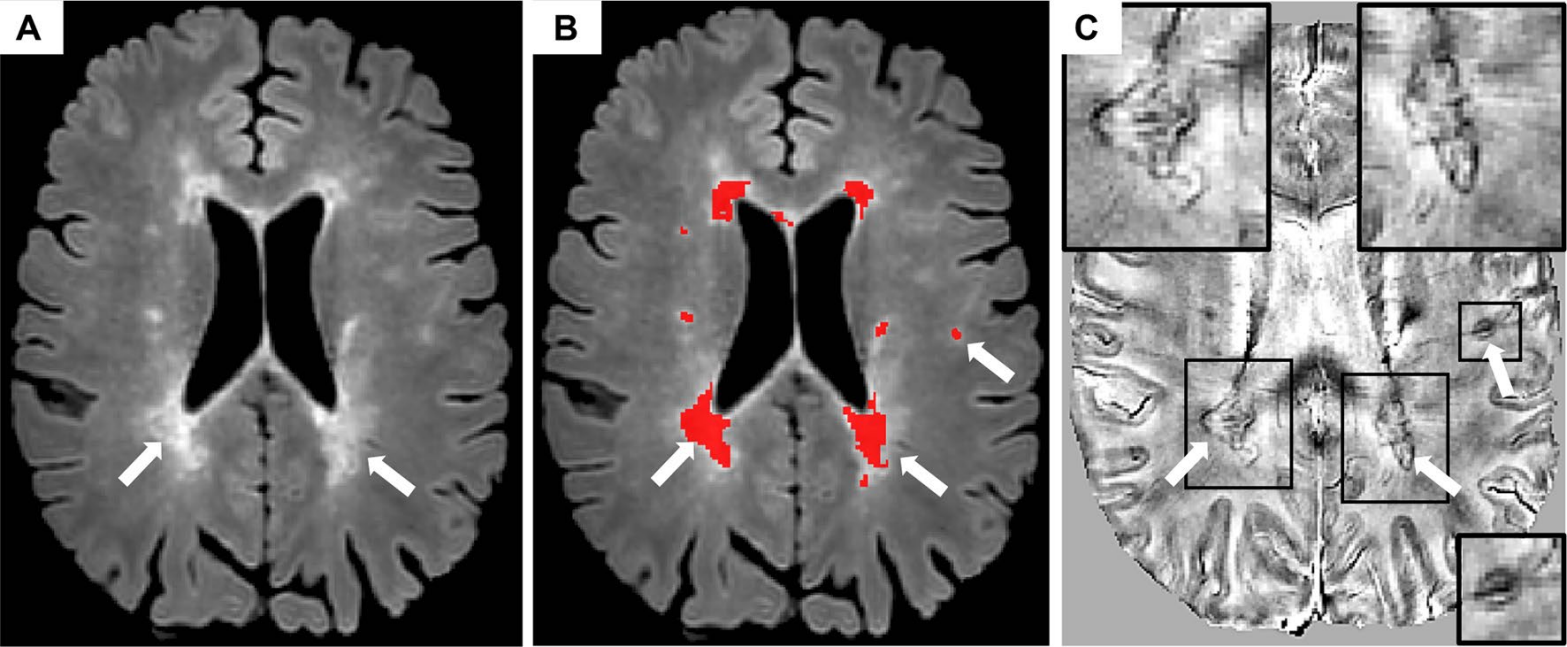
Chronic active lesions. Example of chronic active lesion visualization using susceptibility-weighted MRI. **A** On 3D axial fluid-attenuated inversion recovery sequence in a 48-years-old secondary progressive multiple sclerosis patient (**A**–**B**), two confluent periventricular T2-hyperintense white matter lesions and another subcortical T2-hyperintense white matter lesion (white arrows among red-coded lesion mask) show a hypointense rim on phase image derived from a multi echo gradient-echo T2* sequence (**C**), thus they represent ‘paramagnetic rim lesions’ (PRLs)

Chronic active lesions have been evaluated by looking at susceptibility-weighted MRI scans at high- and ultra-high field [37] (Fig. 2). On these sequences, chronic active lesions show a paramagnetic hypointense rim (i.e., ‘paramagnetic rim lesions’ [PRLs]), which corresponds to peripheral iron-laden microglia/macrophages [37]. Compared to iron negative lesions, PRLs have more severe myelin and axon pathology, a more limited lesional repair and a slow rate of increase in size, at least in the first years after their formation [37, 38].

A higher prevalence and/or number of PRLs have been found to be quite specific and clinically relevant for MS. They have been described in patients with radiologically isolated syndrome (RIS) (12% of all WM lesions, with 61% of RIS patients having ≥ 1 PRLs) [39] and CIS (19.9% of all WM lesions) [40], but not in MS-mimics [40–42], including NMOSD, Susac syndrome, and small-vessel disease.

In a large cohort of subjects with CIS/MS (*n* = 254), MS mimickers (*n* = 91) and old healthy controls (*n* = 271), the identification of ≥ 1 PRLs was the optimal cut-off to distinguish CIS/MS patients from MS mimickers and old healthy controls (specificity = 99.7%, sensitivity = 24.0%, AUC = 0.71, 95% confidence interval [CI] = 0.64–0.78). Of note, the fulfilment of ≥ 1 PRLs or ≥ 4 lesions with CVS improved specificity (90.6%), sensitivity (57.9%) and AUC (0.83, 95% CI = 0.79–0.87) [42].

In CIS patients, the presence of ≥ 1 PRLs and/or the fulfilment of ‘CVS’ criteria (≥ 3 lesions or 40% threshold of lesions with the CVS) predicted MS conversion after 3 years with good sensitivity (70.2–90.4%) and specificity (35.7–85.7%) [40]. Of note, none of the patients who remained CIS after 3 years had any PRLs [40].

Recently, reliable methods have been proposed to automatically detect PRLs [43, 44]. A fully automated method, which applied lesion-level radiomic feature extraction and machine learning on 3D T1-weighted, 3D T2-FLAIR and 3D T2*-phase MRI sequences, showed a strong correlation (*r* = 0.91) with manual rating and an AUC of 0.80 in correctly classifying PRLs in MS patients [44].

By applying a multimodal patch-based convolutional neural network (CNN) (RimNet) on 3D T2*-EPI and 3D T2-FLAIR sequences, a recent study showed a sensitivity (70.6%), specificity (94.9%) and AUC (0.943) comparable to manual rating for PRL identification [43].

By applying a two-branch feature extraction network and a synthetic minority oversampling network (QSMRim-Net) on quantitative susceptibility mapping (QSM) and T2-weighted FLAIR (T2-FLAIR), the proposed methods showed better sensitivity (68%), specificity (98.9%) and accuracy (97.6%) compared to other state-of-the-art methods applied to quantify PRLs on QSM [45].

## Leptomeningeal enhancement

Mild, widespread perivascular inflammatory infiltrates in the meninges are a nearly ubiquitous autopsy finding in all forms of MS and approximately 40–50% of those with SPMS have focal areas of leptomeningeal ectopic lymphoid tissue (“meningeal follicles”) [46]. Gradients of neuronal loss and cortical demyelination emanating from these follicles have been found at autopsy, suggesting a direct causative relationship to cortical lesion formation [47]. Further, intrathecal production of inflammatory cytokines associated with lymphoid follicle activity (i.e., CXCL13, IFN-γ) has been suggested to predict cortical damage on MRI [47, 48].

The potential importance of meningeal inflammation as a contributor to MS pathology led to the search for surrogate imaging biomarkers. Although meningeal pathology in MS does not cause post-contrast enhancement on T1-weighted imaging, it has long been known that this technique is not as sensitive to meningeal disease as post-contrast fluid attenuated inversion recovery (FLAIR) MRI. FLAIR is > 10 times more sensitive to small concentrations of Gd in CSF and is more sensitive to the presence of leptomeningeal enhancement (LME) in conditions such as leptomeningeal carcinomatosis and infectious meningitis.

Although, at present, no study directly compared 3T and 7T images, a recent meta-analysis showed that, in MS patients, higher LME-proportions were found in studies imaging at 7T (0.79 [95%-CI 0.64–0.89]) compared to lower field strengths (0.21 [95% CI 0.15–0.29], *p* < 0.001) [49].

**Fig. 3:**
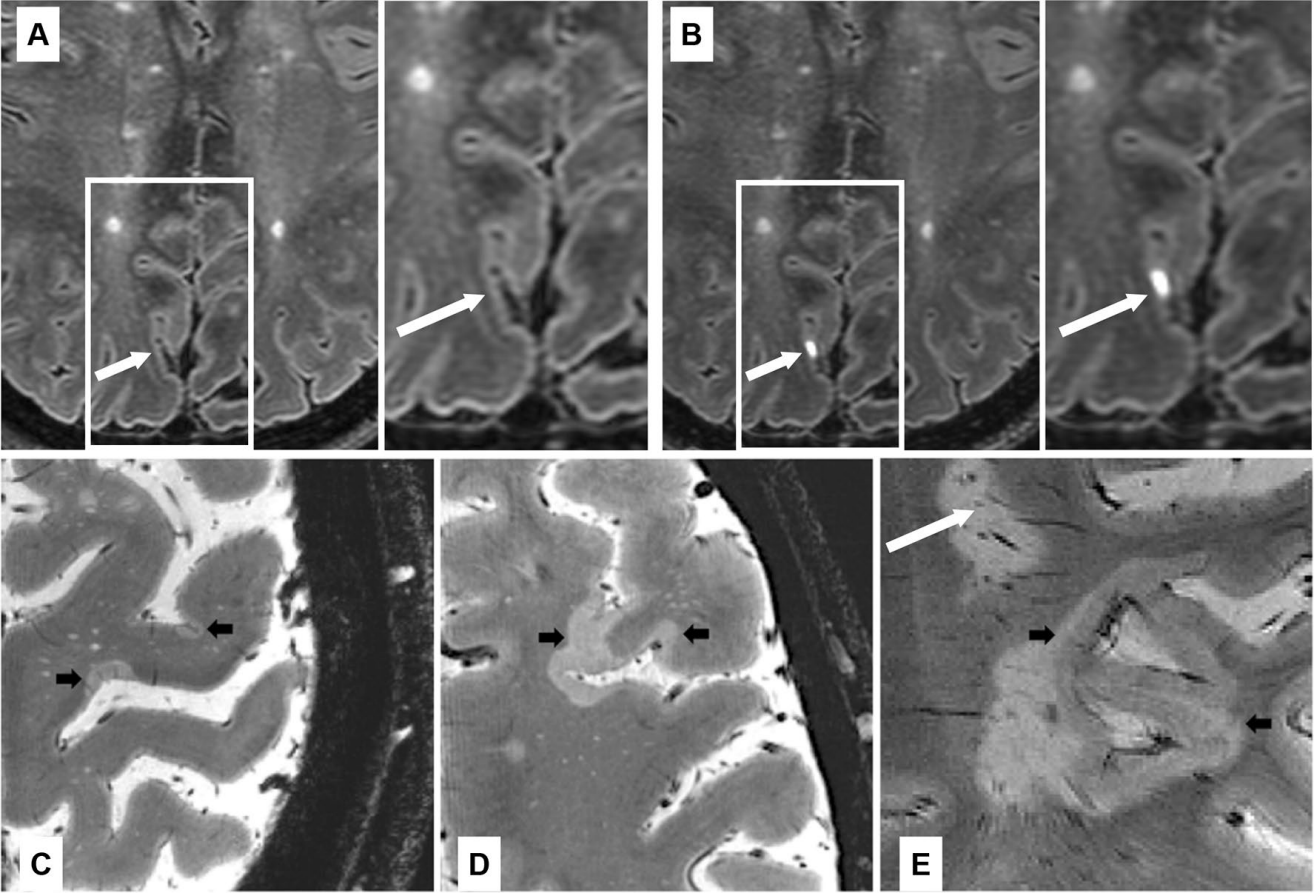
Leptomeningeal enhancement, cortical lesions and subpial demyelination. **A**, **B** Example of 7T FLAIR MRI of the brain in a 49-years-old woman with relapsing–remitting multiple sclerosis before (**A**) and after (**B**) the administration of gadolinium-based intravenous contrast agent. A focus of post-contrast pial/subarachnoid enhancement highlighted by white arrow. Examples of focal (**C**) or more extensive (**D**, **E**) subpial multiple sclerosis lesions (black arrows) with expansion within white matter/confluence with a juxtacortical lesion (**E**) in patients with multiple sclerosis as seen on ultrahigh resolution T2* gradient echo images at 7 Tesla. A white matter lesion is also visible in (**E**) (white arrow). Abbreviations: *FLAIR* fluid-attenuated inversion recovery, *MRI* magnetic resonance imaging

With this knowledge, post-contrast FLAIR MRI protocols were tested as a potential surrogate biomarker of meningeal inflammation in MS (Fig. 3). A large study of delayed-acquisition, 3D FLAIR on 3T MRI showed Gd deposition in the leptomeningeal space in 25% of MS patients compared to 2.7% of controls [50]. Two patients from this study later went to autopsy and cellular inflammatory infiltrates were found in regions of leptomeninges correlating to locations of enhancing foci during life, supporting LME as a potential surrogate of meningeal inflammation in MS.

However, the finding of LME on post-contrast FLAIR is not specific to MS. A 7T study described two patterns of LME: “nodular” (i.e., discrete, spherical nodules at the pial surface or subarachnoid space) and “spread/fill” (appearance of contrast spread through the local subarachnoid space). Nodular foci were present in 15 of 29 (51%) MS patients and in 2 out of 3 (67%) of healthy controls, whereas spread/fill foci were present in 22 of 29 (76%) MS patients [51]. In another study, LME was found in 5 out of 66 healthy controls (5.7%) [52].

Brain LME, either as a single or multiple nodular foci, is also frequently seen in patients with other inflammatory neurologic conditions (18/51 cases [35.3%]), such as NMOSD (6/11 cases [54.5%]) and MOGAD (3/11 cases [27.3%]), non-inflammatory neurologic diseases (i.e., including small vessel disease, migraine, neurodegenerative diseases, and compressive myelopathy) (3/38 cases [7.9%]), reversible cerebrovascular constriction syndrome (69/182 cases [37.9%]), and infectious conditions such as human T-lymphotropic virus (HTLV) infection (17/38 cases [44.7%]) and human immunodeficiency virus (HIV) (13/61 cases [21.3%]) [52–55].

A recent meta-analysis evaluated the presence of LME in neoplastic neurological (*n* = 2392 cases), neuroinfectious (*n* = 1890 cases) and primary neuro-inflammatory diseases (*n* = 4038) [49]. The LME proportions for these disease classes were 0.47 (95% confidence interval [CI] = 0.37;0.57), 0.59 (95% CI = 0.47;0.69), and 0.26 (95% CI = 0.20;0.35), respectively. In a subgroup analysis for MS (*n* = 1605 cases), LME proportion was 0.30 (95% CI = 0.21;0.42) with lower proportions in RRMS (0.19 [95% CI = 0.13;0.27]) compared to progressive MS (0.39 [95% CI = 0.30;0.49], *p* = 0.002) [49].

Accordingly, brain LME on post-contrast FLAIR lacks the specificity necessary for use as a diagnostic biomarker in MS. Moreover, although LME could become a surrogate of meningeal inflammation in MS, a direct relationship is not yet established. Recent work showed that post-contrast FLAIR MRI may also directly visualize brain lymphatic vessels and glymphatic clearance [55, 56], suggesting that focal blood-meningeal barrier breakdown may not be the only source of enhancement.

## Subpial demyelination

Cortical demyelinating lesions are an established pathological feature of MS, can develop from the earliest disease stages and represent one of the main substrates of disease progression [4]. Detection of cortical lesions by MRI can be used in clinic as evidence of DIS to aid the diagnosis of MS. Clinical MRI protocols at 1.5 and 3T are, however, hampered in their ability to visualize and characterize the full extent of cortical demyelination in vivo and most of the knowledge on cortical MS lesions derives from post-mortem studies. Neuropathological examinations have identified different types of cortical lesions in MS based on their location within the cortical laminae: type I leukocortical lesions located at cortical-subcortical junction; type II, intracortical plaques, very small lesions completely enclosed in the cortex without reaching its margins; type III–IV subpial lesions, extending downwards from the juxtameningeal pial surface through different cortical laminae or across the full cortical depth. Subpial lesions represent the most common type of cortical MS lesions and is vastly underdetected by conventional MRI.

Ultra-high field 7T MRI provides several advantages over lower field MR strength in imaging cortical lesions in MS (Fig. 3). The increase in signal-to-noise ratio (SNR) achieved at ultra-high field can be translated into a resolution in the sub-millimeter range and exquisite contrast for imaging details within both gray matter (GM) and WM. Although we are still far from revealing the true number of cortical lesions in vivo, correlative histopathological 7T MRI assessments have shown ultra-high field MRI can more than double cortical lesion detection relative to 3T MRI [57]. The gain in resolution and delineation across cortical layers allows the identification at 7T of same cortical lesion subtypes observed in histopathological examinations [58, 59].

Increased cortical lesion detection achieved at 7T is of high interest because subpial lesions are a typical finding in MS [60] and their visualization could, therefore, improve the MS diagnostic sensitivity and specificity. However, cortical lesions and subpial demyelination are also not fully specific for MS since they have been also described in patients with acute disseminated encephalomyelitis (ADEM) [60] and MOGAD [53].

Different pulse sequences have been optimized at 7T, including T2*-weighted gradient-echo, T2-weighted, FLAIR, double inversion recovery (DIR), and T1-weighted magnetization-prepared rapid-acquisition gradient-echo (MPRAGE/MP2RAGE), to image cortical lesions in different MS stages [57–59, 61]. As different sequences seem to favor the identification of different cortical lesion subtypes (e.g., T2*-weighted gradient-echo for subpial lesion detection [57]; MP2RAGE for small intracortical lesions) [62], the information provided by different contrasts is frequently complementary and, when used jointly, can only increase the definition of cortical lesions. Cortical lesion identification and segmentation, however, strongly rely on readers’ training and experience, and the attempts to automatize this process are still far from a clinically acceptable performance [63, 64]. Further improvements in the standardization of both acquisitions and processing methods across Centers are still needed for a translation of these techniques in clinic.

The increase in accuracy of cortical lesion detection at 7T could also be used to guide selective characterization of the different pathological components of cortical lesions, including demyelination and inflammation, in MS through complementary techniques such as PET, which are able to estimate the distribution of specific molecules or processes of interest. Investigations combining 7T MRI and PET imaging of neuroinflammation have revealed an association between cortical demyelination and glial activation [65] as well as heterogeneity in levels of inflammation within cortical lesions [66]. This approach could be used in the future to stage cortical lesion inflammatory activity in vivo and, potentially, to improve MS diagnosis.

## The contribution of AI

Recent improvements in technologies and the availability of large amount of data have promoted the application of AI algorithms for the diagnostic-work up of MS [7]. Using CNN, a model of deep-learning (DL) tool able to automatically select the best problem-solving features, recent AI studies were able to discriminate between MS patients and HC with an accuracy between 70.2 and 98.8% from T2-weighted [67, 68], FLAIR [69], or susceptibility-weighted MRI sequences [70]. Machine learning (ML) algorithms, used to learn from specific predefined data features and then make decisions and trained on quantitative [71–73], diffusion-weighted [74], and resting state functional [74, 75] MRI sequences, were also able to correctly identify MS patients with an accuracy ranging from 83.7 to 90.0%.

AI algorithms can contribute discriminating MS from mimics. Using random forest, a supervised learning algorithm, on a set of brain GM imaging measures, lower thalamic volume together with other measures of brain GM volumes and cortical thickness obtained from high-resolution T1-weighted sequences were found to discriminate NMOSD from MS with an accuracy of 74% [76]. A multiparametric approach including data from FLAIR, diffusion-tensor imaging, resting state functional MRI plus clinical and neuropsychological information improved the diagnostic performance to 88% [77].

A CNN algorithm applied on brain FLAIR sequences and patients’ clinical information (age at disease onset, age at the time of MRI, disease duration, time from the last relapse) discriminated AQP4-positive NMOSD from MS patients with an accuracy similar to expert neurologists (accuracy = 71.1% vs 65.9–60.7%), but higher reliability (human intra-rater reliability of 0.47–0.50) [78]. In another study, a CNN algorithm trained on brain FLAIR and T1-weighted sequences showed a higher accuracy compared to that of two expert neuroradiologists in correctly discriminate MS (98.8% *vs* 72.8–81.8%) from other MS mimics, including NMOSD (88.6 vs 4.4%), migraine (92.2% vs 53–64.8%) and CNS vasculitis (92.1% vs 45.5–54.6%) [79]. Other ML and DL algorithms trained on conventional MRI sequences (FLAIR, PD, T2-weighted and T1-weighted) or proton magnetic resonance spectroscopy were also able to discriminate between MS, non-inflammatory WM disorders (hereditary diffuse leukodystrophy with spheroids and cerebral microangiopathy) [80, 81], or low-grade brain tumors [82].

ML algorithms applied on baseline demographic (age, sex), clinical (EDSS score and type of onset), and brain MRI features (including WM lesion count, radiomic features, regional GM atrophy and cortical thickness) predicted also conversion from CIS to MS with an average accuracy between 71.4 and 92.9% at 1 year [83, 84], between 67.6 and 70.4% at 2 years [85] and between 68.0 and 85.0% at 3 years follow-up [83, 86].

Although AI methods have been showing increasingly promising results in MS diagnostic work-up, several limitations should be taken into account. First, it may be challenging to interpret neural network decisions. For instance, AI methods (especially DL algorithms) may follow “shortcut” strategies, which, while superficially successful (i.e., differentiation between MS patients from HC based on the presence/absence of lesions), typically fail under different circumstances (i.e., differentiation between MS patients from others with WM lesions due to cerebral small vessel disease). Moreover, possible selection bias and overfitting may overestimate the performance of AI algorithms. AI approaches also need large datasets, which may be challenging to obtain due to system availability, high costs, and heterogeneous acquisition methodologies. To overcome such limitation and to allow their use in the clinical scenario, AI algorithms should be cross-validated in multicenter, prospective and longitudinal real-world cohorts, to overcome challenges due to variability of image acquisition parameters and scanner models and the presence of heterogeneous distribution of data between an algorithm's training dataset and validation. Furthermore, AI algorithms should be integrated into existing information technology infrastructures and the access to the required computing power should be guaranteed. Finally, guidelines that ensure reliability and validity of findings obtained from AI approaches, and standard thresholds for the accuracy of models required for publication, which are lacking at present.

## Conclusions

Accurate criteria in the diagnostic work-up of patients with a suspicion of MS are crucial not only to enable an early diagnosis, thus allowing treatment to start sooner, but also to minimize the risk of misdiagnosis and overdiagnosis.

The 2017 McDonald criteria are validated and evidence-based criteria that show high sensitivity and accuracy in predicting the occurrence of a second clinical attack, simplify the clinical use of MRI criteria, and allow an earlier diagnosis and treatment of MS. Some concerns have been raised due to their low specificity. However, their application is recommended only after alternative diagnoses have been carefully excluded.

To further improve the diagnostic process, novel candidate imaging biomarkers, such as CVS and chronic active lesions have been proposed to increase the specificity of MS diagnostic criteria, thus reducing the risk of misdiagnosis (Table 3).

**Table 3:** Summary of newly proposed MRI markers for the diagnostic work-up of MS

MRI marker	MRI sequence(s)	Pathological substrate	Specificity in differential diagnosis	Prediction of MS conversion	Overall contribution for early MS diagnosis	Prediction of disability progression	Feasibility in the clinical setting	Associations with brain damage
Central vein sign	SWI (T2*-weighted 3D-EPI)	Perivenular inflammation	+ + +	+	+ + +	?	+ + +	?
Paramagnetic rim lesions	SWI (T2*-weighted 3D-EPI)	Chronic active lesions	+ + +	+ +	+ +	+ +	+ + +	+ +
Leptomeningeal enhancement	Post-Gd T2-weighted FLAIR	Meningeal inflammation	+	?	±	+	+ +	+
Cortical lesions	DIR; 3D T1-weighted MPRAGE/MP2RAGE PSIR	Cortical demyelination	+ + +	+ +	+ +	+ + +	+ +	+ +
Subpial demyelination	T2*-weighted gradient-echo	Cortical demyelination	+ + +	+ +	?	+ + +	+	+ +

*3D-EPI* three-dimensional echo-planar imaging, *DIR* double inversion recovery, *FLAIR* fluid-attenuated inversion recovery, *Gd* gadolinium, *MP2RAGE* magnetization prepared 2 rapid gradient echoes, *MPRAGE* magnetization-prepared rapid gradient echo, *MRI* magnetic resonance imaging, *MS* multiple sclerosis, *PSIR* phase-sensitive inversion recovery, *SWI* susceptibility weighted imaging

However, they should be further validated and standardized before being implemented in the clinical setting. In particular, future studies should ascertain their role to exclude alternative diagnoses but also to diagnose MS in patients with both relapse- or progressive-onsets.

Subpial demyelination is highly specific for MS but hardly visible at standard field strengths (i.e., 1.5 and 3.0 Tesla scanners), whereas leptomeningeal enhancement is not MS-specific, since it can be detected in other inflammatory CNS diseases [52] and with ageing [87].

In the near future, AI approaches may represent a complimentary tool for neurologists and neuroradiologists. Beside visual-pattern recognition performed by experienced clinicians of lesional features typical for MS, which may be time-consuming and hardly reproducible, AI may allow to identify textures and patterns on MRI sequences that are beyond the human perception and may further improve the diagnostic work-up and patients’ classification.

## Data Availability

This is a review article. Publications cited in the manuscript are generally available online, as referenced in the reference section.
